# Determination of protein markers of inflammasome formation and ferroptosis in thyroid cancer based on gender

**DOI:** 10.7150/jca.128886

**Published:** 2026-04-16

**Authors:** Raúl Díaz-Pedrero, Oscar Fraile-Martinez, Cielo García-Montero, Diego Liviu Boaru, Patricia De Castro-Martinez, Diego De Leon-Oliva, Laura López-Gonzalez, Beatriz García-González, Isabel Pérez-González, Majd N. Michael Alhaddadin, Silvestra Barrena-Blázquez, Connie Ferrara-Coppola, Ana María Villar-Carrillo, Laura Gutierrez-Gonzalez, Miguel Pascual-Casero, Félix Mañez, Tomás Ratia Giménez, Inmaculada Lasa-Unzúe, Manuel Díez Alonso, Alberto Gutiérrez-Calvo, Luis G Guijarro, Melchor Álvarez-Mon, Montserrat Chao Crecente, Miguel A Saez, Miguel A Ortega

**Affiliations:** 1 Department of General and Digestive Surgery, Príncipe de Asturias, University Hospital, 28805 Alcala de Henares, Spain.; 2 Department of Surgery, Medical and Social Sciences, Faculty of Medicine and Health Sciences, University of Alcalá, 28801 Alcala de Henares, Spain.; 3 Ramón y Cajal Institute of Sanitary Research (IRYCIS), 28034 Madrid, Spain.; 4 Department of Medicine and Medical Specialities, Faculty of Medicine and Health Sciences, Network Bi-omedical Research Center for Liver and Digestive Diseases (CIBEREHD), University of Alcalá, 28801 Alcala de Henares, Spain.; 5 Department of Nursing and Physiotherapy, Faculty of Medicine and Health Sciences, University of Alcalá, 28801 Alcala de Henares, Spain.; 6 Pathological Anatomy Service, Central University Hospital of Defence-UAH Madrid, 28801 Alcala de He-nares, Spain.; 7 Unit of Biochemistry and Molecular Biology (CIBEREHD), Department of System Biology, University of Al-calá, 28801 Alcalá de Henares, Spain.; 8 Immune System Diseases-Rheumatology, Oncology Service an Internal Medicine (CIBEREHD), University Hospital Príncipe de Asturias, 28806 Alcala de Henares, Spain.; 9 Pathological Anatomy Service of the “Severo Ochoa” University Hospital in Leganés, Spain.

**Keywords:** papillary thyroid carcinoma (PTC), ferroptosis, NLRP3 inflammasome, IRS-4, KLOTHO

## Abstract

Papillary thyroid carcinoma (PTC) is the most common subtype of thyroid cancer, with a globally increasing incidence. The disease exhibits significant sex-related clinical and biological differences: while incidence is approximately three times higher in women, men face a greater risk of lymph node metastasis, recurrence, and mortality. This gender disparity may be associated with hormonal factors, particularly estrogens, which are known to modulate cellular proliferation, invasion, migration, and adhesion in thyroid tumor cells. However, the molecular pathways underlying these effects remain poorly understood. In this context, the hallmarks of cancer proposed by Hanahan—such as sustained proliferative signaling, evasion of cell death, and reprogramming of the tumor microenvironment—provide a valuable framework to investigate sex-based disparities. This study assessed, via immunohistochemistry, the expression of molecular markers related to ferroptosis (GPX4, ALOX5, ACSL4, TFRC), the NLRP3 inflammasome and its components (NLRP3, ASC, caspase-1, caspase-5, caspase-8, IL-1β, and IL-18), proliferation (IRS-4), and tumor suppression (KLOTHO) in PTC samples from 25 men and 25 women. Our results demonstrated increased expression of ferroptosis-related markers, components of the NLRP3 inflammasome, and IRS-4 in female-derived samples, whereas male samples exhibited higher KLOTHO expression levels. These findings support the hypothesis of a molecular basis for sexual dimorphism in PTC and highlight the need for further research with larger cohorts and mechanistic approaches to elucidate these pathways and their therapeutic potential.

## 1. Introduction

Thyroid cancer is the most common endocrine neoplasm, and its incidence has steadily increased worldwide in recent decades 1. According to data from the International Agency for Research on Cancer (IARC), collected in the GLOBOCAN 2022 database, thyroid cancer ranks seventh in terms of global incidence of all types of cancer, ranking as the fifth most common tumor in women and thirteenth in men 2. Despite its high incidence, thyroid cancer does not have a high mortality rate: the five-year survival rate exceeds 98%, with an estimated annual mortality rate of approximately 0.5 per 100,000 inhabitants 3. The average age of diagnosis is around 51, with the condition being most common between the ages of 45 and 64 1.

From a histopathological point of view, thyroid cancer is mainly classified into differentiated forms—papillary and follicular carcinoma—poorly differentiated forms, which include medullary carcinoma, and finally, anaplastic carcinoma. Of all these, papillary thyroid carcinoma (PTC) is the most common subtype, accounting for between 80% and 85% of cases 4. This high prevalence has led to increased interest in characterizing its molecular mechanisms and prognostic factors.

Scientific evidence has shown that TPC presents significant clinical and biological differences between men and women 5,6,7 Although the incidence is approximately three times higher in women, men tend to present with more aggressive disease with a higher risk of lymph node metastasis, recurrence, and mortality 7,8. This gender disparity could be related to hormonal factors, especially estrogens, which influence proliferation, invasion, migration, and cell adhesion in thyroid tumor cells 5,9. Likewise, during pregnancy, increases in estrogen and chorionic gonadotropin have been associated with accelerated growth of thyroid nodules 10. Understanding the biological basis and differences between sexes could have important clinical implications for the management of TPC, as reflected in the scientific literature 6. However, the molecular mechanisms that differentiate men and women are still not fully understood.

In 2000, Hanahan and Weinberg proposed the concept of “hallmarks of cancer,” a set of functional characteristics acquired by tumor cells during carcinogenesis 11. This proposal has been progressively expanded until, in its most recent revision in 2022, fourteen hallmarks were established, including: sustained proliferative signaling, evasion of cell death, invasion and metastasis, genomic instability, cellular plasticity, and alterations in interaction with the immune system and the tumor microenvironment 12. Although many tumors share these attributes, the way in which each type of cancer acquires them varies, underscoring the importance of studying them in specific contexts such as the TPC.

Ferroptosis is a form of iron-dependent cell death characterized by the accumulation of lipid peroxides that irreversibly damage the cell membrane 13. Its dysfunction has been linked to progression and metastasis in various tumors, including TPC 14,15. Markers such as GPX4, which inhibits ferroptosis by reducing lipid peroxides; ALOX5 and ACSL4, which promote its formation; and TFRC, involved in iron uptake, have demonstrated their role in TPC carcinogenesis, showing their potential value as biomarkers 13,16,17. Similarly, the influence of gender on the expression levels of these components is beginning to be elucidated in recent studies 18.

The NLRP3 inflammasome is a multiprotein complex of the innate immune system formed by NLRP3, ASC, and caspase-1, whose activation promotes the maturation of inflammatory cytokines such as IL-1β and IL-18 19. There are also non-canonical pathways involved in the activation of this complex, in which caspases 5 and 8 participate 20,21. The NLRP3 inflammasome can act as a tumor promoter or suppressor depending on the tissue context 22. This, together with the fact that the immune response and the NLRP3 inflammasome can be differentially modulated by sex hormones, makes these markers a target of interest for understanding the impact of sex on the inflammatory tumor response in TPC 23.

KLOTHO, meanwhile, is a protein with anti-aging functions that exerts antitumor effects, mainly by inhibiting oncogenic pathways such as PI3K/AKT, WNT, and β-catenin or by regulating processes such as oxidative stress 24-27. In various types of cancer, including TPC, KLOTHO underexpression is associated with a worse prognosis 28. Furthermore, KLOTHO shows possible differential expression between sexes and could be related to the higher incidence of TPC in women, making it a relevant target for studying this disparity 29

Finally, insulin receptor substrate 4 (IRS-4) is an intracellular signaling adapter protein activated by insulin receptors and insulin-like growth factor type 1 (IGF1R). Through pathways such as PI3K/AKT, it participates in cell proliferation and survival^30^. In recent years, several studies have pointed to the emerging role of IRS-4 in oncogenesis, linking it to the constitutive activation of proliferative pathways even in the absence of extracellular stimuli 30 In addition, the differential role of IRS-4 between sexes has been suggested, as demonstrated in animal models 31, highlighting the importance of studying IRS-4 in the context of TPC and its possible interaction with the sex-associated hormonal environment.

Given the growing interest in understanding the molecular mechanisms underlying sexual dimorphism in TPC, and in light of the global increase in its incidence, it is essential to study markers linked to the different hallmarks of cancer. Therefore, in the present study, we analyzed the immunohistochemical expression of ferroptosis (GPX4, ALOX5, ACSL4, TFRC), the NLRP3 inflammasome, KLOTHO, and IRS-4 in PC samples from men and women, with the aim of identifying possible differences in expression that may contribute to explaining the clinical-biological variability between sexes.

## 3. Patients and Methods

### 3.1 Study design and ethical issues

A retrospective observational study was designed based on a cohort of 50 patients with papillary thyroid carcinoma who underwent thyroid resection. The sample included 25 men (median age: 65 years) and 25 women (median age: 62 years). Data were collected on relevant predictive variables, including demographic characteristics (sex and age) and histopathological characteristics of 13 selected biomarkers: ACSL4, ALOX-5, ASC, CASPASE-1, CASPASE-5, CASPASE-8, GPX4, IL-1β, IL-18, IRS-4, KLOTHO, NLRP3, TFRC. All variables included in the analysis were complete, with no missing data.

The study strictly adhered to fundamental ethical principles, including autonomy, beneficence, non-maleficence, and distributive justice. It was conducted in accordance with Good Clinical Practice guidelines and the ethical standards outlined in the latest Declaration of Helsinki (2013) and the Oviedo Convention (1997). All data and information collected were handled in compliance with current data protection legislation, including Organic Law 3/2018, of December 5, on Personal Data Protection and Guarantee of Digital Rights, as well as Regulation (EU) 2016/679.

### 3.2 Participants and sample collection

The diagnosis was made based on the WHO 2022 classification of thyroid neoplasms on thyroid cancer, which provides updated information on the analysis and treatment of PTC 32

Thyroid cancer samples were stored in minimal essential medium (MEM; Thermo Fisher Scientific, Inc., Waltham, MA, USA) supplemented with 1% antibiotic/antifungal (streptomycin, amphotericin B, and penicillin; Thermo Fisher Scientific, Inc.). Sample processing was performed under sterile conditions in a class II laminar flow hood (Telstar AV 30/70 Müller 220 V 50 MHz; Telstar; Azbil Corporation, Chiyoda-ku, Tokyo, Japan).

To remove erythrocytes, samples stored in MEM were washed and rehydrated five times with antibiotic-free MEM. They were then cut into 2 cm sections using a new sterile scalpel and fixed in an F13 solution (composed of 60% ethanol, 20% methanol, 13% distilled water, and 7% polyethylene glycol), following previously established protocols 33.

The samples were embedded in paraffin molds, sectioned into 5 µm slices using an HM 350 S rotary microtome (Thermo Fisher Scientific, Inc., Waltham, MA, USA), and transferred to slides treated with 10% poly-L-lysine after being placed in a warm water bath. These preparations were then treated for histological studies.

### 3.3 Immunohistochemical techniques

The detection of antigen-antibody reactions used the avidin-biotin complex (ABC) method, employing avidin-peroxidase according to the protocols established by Ortega *et al*. 34. Table 1 shows the characteristics of each of the antibodies used in this study, the dilutions used, the supplier, and the protocol specifications.

After incubation with the primary antibody for 1 hour and 30 minutes, the samples were incubated overnight with 3% BSA blocker (catalog #37525; Thermo Fisher Scientific, Inc., Waltham, MA, USA) and PBS at 4°C. Subsequently, the samples were incubated with biotin-conjugated secondary antibody diluted in PBS for 90 minutes at room temperature (RT). Rabbit IgG, diluted 1/300 (RG-96; Sigma-Aldrich, St. Louis, MI, USA), goat IgG, diluted 1/100 (GT-34/B3148; Sigma-Aldrich), and mouse IgG, diluted 1/300 (F2-3148; Sigma-Aldrich), were used), goat IgG diluted 1/100 (GT-34/B3148; Sigma-Aldrich), and mouse IgG diluted 1/300 (F2012/045K6072; Sigma-Aldrich) were used.

After this, the samples were incubated with ExtrAvidin® peroxidase conjugate (Sigma-Aldrich; Merck KGaA, Darmstadt, Germany) for 60 minutes at RT (diluted 1:200 with PBS). Protein expression levels were assessed using a diaminobenzidine (DAB) chromogenic substrate kit (cat. no. SK-4100; Maravai LifeSciences, San Diego, CA, USA), freshly prepared prior to use. The peroxidase chromogenic substrate was applied for 15 minutes at RT, allowing the development of brown staining indicative of protein expression.

For each protein, negative control sections were included, where incubation with primary antibody was replaced with a blocking solution (PBS). Carazzi hematoxylin staining was performed for 15 minutes at RT to provide contrast in all tissues.

### 3.4 Histopathological analyses and statistical tests

Using an AxioCam HRc digital camera and a Zeiss Axiophot optical microscope (Carl Zeiss, Oberkochen, Germany), two independent pathologists observed tissue sections from each sample for the markers explored. The interpretation of histological expression was performed using the immunoreactivity scoring system (IRS). The scores obtained in this study are coded as 1, 2, and 3, corresponding to low, medium, and high expression, respectively, as defined in previous studies 35

All statistical analyses were performed using RStudio (version 4.1.3). As an initial step, the distribution of the data was evaluated using the Shapiro-Wilk test, confirming its non-normality; consequently, non-parametric tests were applied throughout the study.

First, a description of the cohort was performed using the *DescribeBy* function, with the aim of obtaining the median age for each group according to sex. Next, violin plots were created to visualize the distribution of biomarkers according to sex, and the Mann-Whitney U test was applied to compare the differences between groups.

Finally, we generated heatmap-type correlograms, constructing a matrix of Spearman's correlation coefficients for each sex, based on the IRS scores. These representations were made using the corrplot package. The p-values were adjusted using the false discovery rate (FDR) method.

## 4. Results

### 4.1 Women with papillary thyroid carcinoma have a molecular profile characterized by overexpression of IRS-4 and reduced levels of KLOTHO compared to men

**Figure 1:**
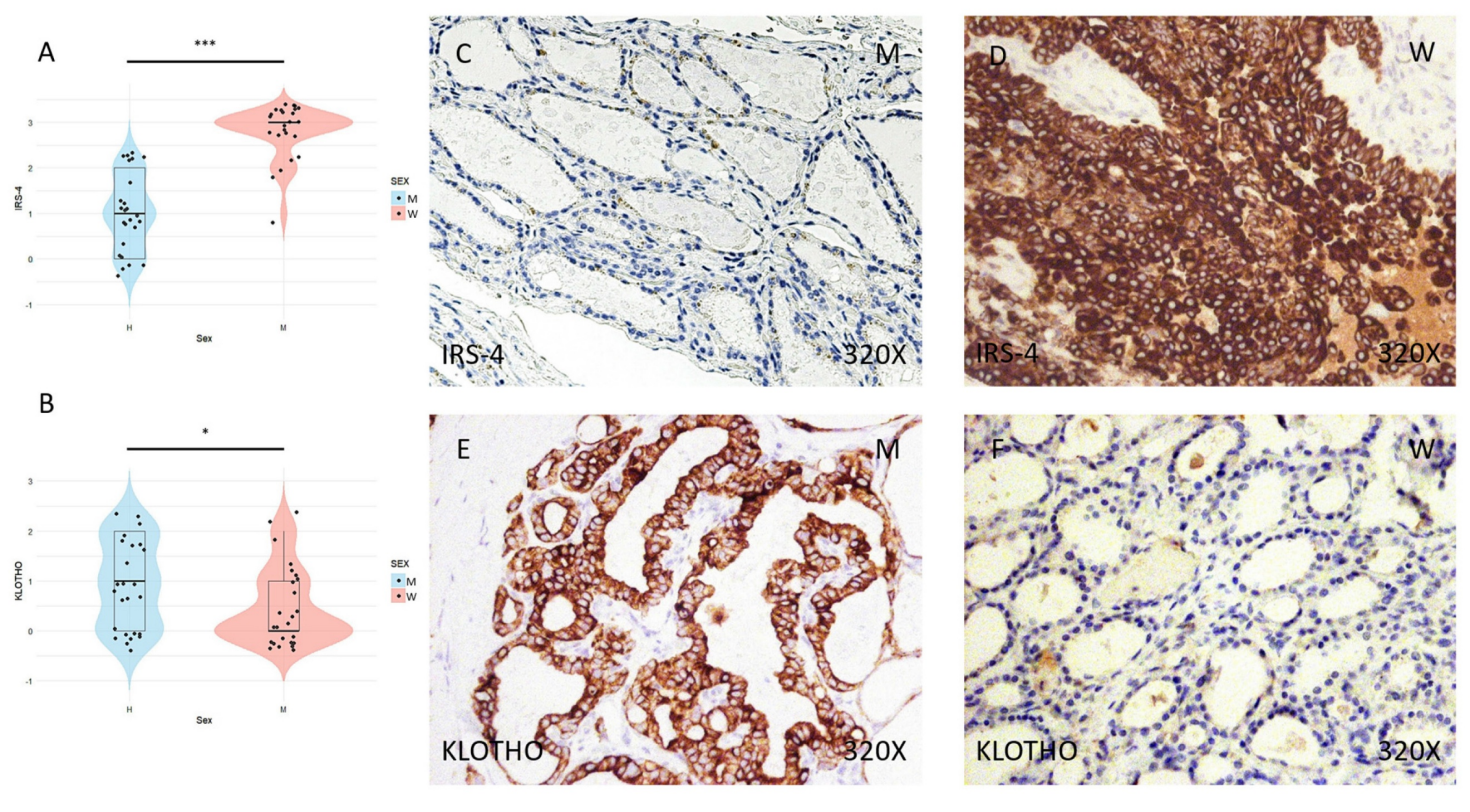
** (A)** Distribution of IRS-4 expression (median in women: 3; median in men: 1; p value=6.258e-09) in papillary thyroid carcinoma samples according to sex, represented by a violin plot. **(B)** Distribution of KLOTHO biomarker expression (median in women: 0; median in men: 1; p value=0.0364) in papillary thyroid carcinoma samples according to sex, represented by a violin plot. **(C)** and **(D)** Images of immunohistochemical expression of IRS-4, showing increased protein expression in women. **(E)** and** (F)** Images of KLOTHO expression, showing increased expression in men compared to women. 320x magnification. Significance levels: p<0.05 (*), p<0.01 (**), p<0.001(***).

Immunohistochemical analysis of the samples shows higher expression of the IRS-4 proliferation protein in the tumor tissue of women than in that of men (**Figure 1C-D**), a difference that was significant in the subsequent statistical analysis (median in women: 3; median in men: 1; p-value = 6.258e-09) (**Figure 1A**). In contrast, the KLOTHO aging marker shows higher expression in men than in women (median in women: 0; median in men: 1; p-value = 0.0364), as evidenced by immunohistochemical study (**Figure 1E-F**) and subsequent examination of the results (**Figure 1B**).

### 4.2 Women with papillary thyroid carcinoma show significant overexpression of ferroptosis markers

**Figure 2:**
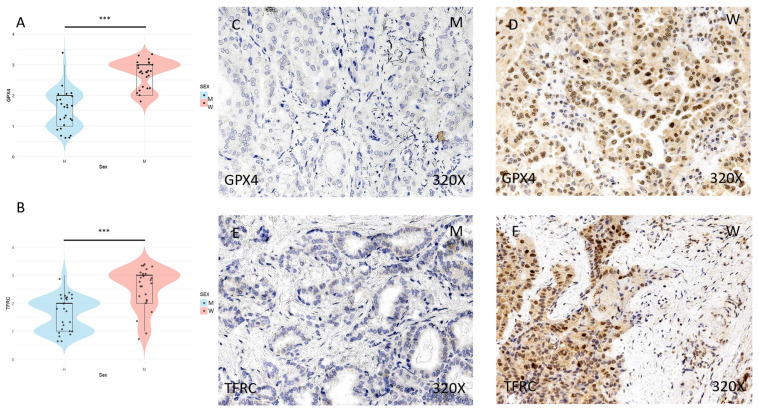
** (A)** and **(B)** Distribution of the expression of ferroptosis markers GPX4 (median in women: 3; median in men: 2; p value=1.415e-07) and TFRC (median in women: 3; median in men: 2; p value=3.517e-05) in papillary thyroid carcinoma samples according to sex, represented by a violin plot. **(C-F)** Images of immunohistochemical expression of GPX4 and TFRC, showing increased protein expression in women. 320x magnification. Significance levels: p<0.05 (*), p<0.01 (**), p<0.001(***).

**Figure 3:**
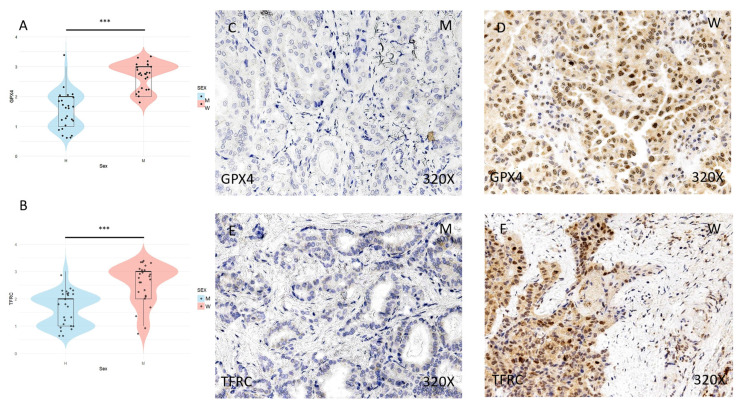
** (A)** and **(B)** Distribution of the expression of ferroptosis markers ACSL-4 (median in women: 3; median in men: 1; p value=5.279e-06) and ALOX-5 (median in women: 3; median in men: 1; p value=5.998e-09) in papillary thyroid carcinoma samples by sex, represented by a violin plot. **(C-F)** Images of the immunohistochemical expression of ACSL-4 and ALOX-5, showing an increase in protein expression in women. 320x magnification. Significance levels: p<0.05 (*), p<0.01 (**), p<0.001(***).

The expression of ferroptosis markers GPX4, TFRC, ACSL4, and ALOX-5 was studied using immunohistochemical techniques, showing an increase in tumor tissue in women compared to men (**Figures 2 and 3** (**C-F**)). After quantification, this difference was significant in all cases, namely GPX4 (median in women: 3; median in men: 2; p-value=1.415e-07), TFRC (median in women: 3; median in men: 2; p-value=3.517e-05), ACSL-4 (median in women: 3; median in men: 1; p value=5.279e-06), and ALOX-5 (median in women: 3; median in men: 1; p value=5.998e-09) (**Figures 2 and 3** (**A-B**)).

### 4.3 Women with papillary thyroid carcinoma show a significant increase in NLRP3 inflammasome markers

**Figure 4:**
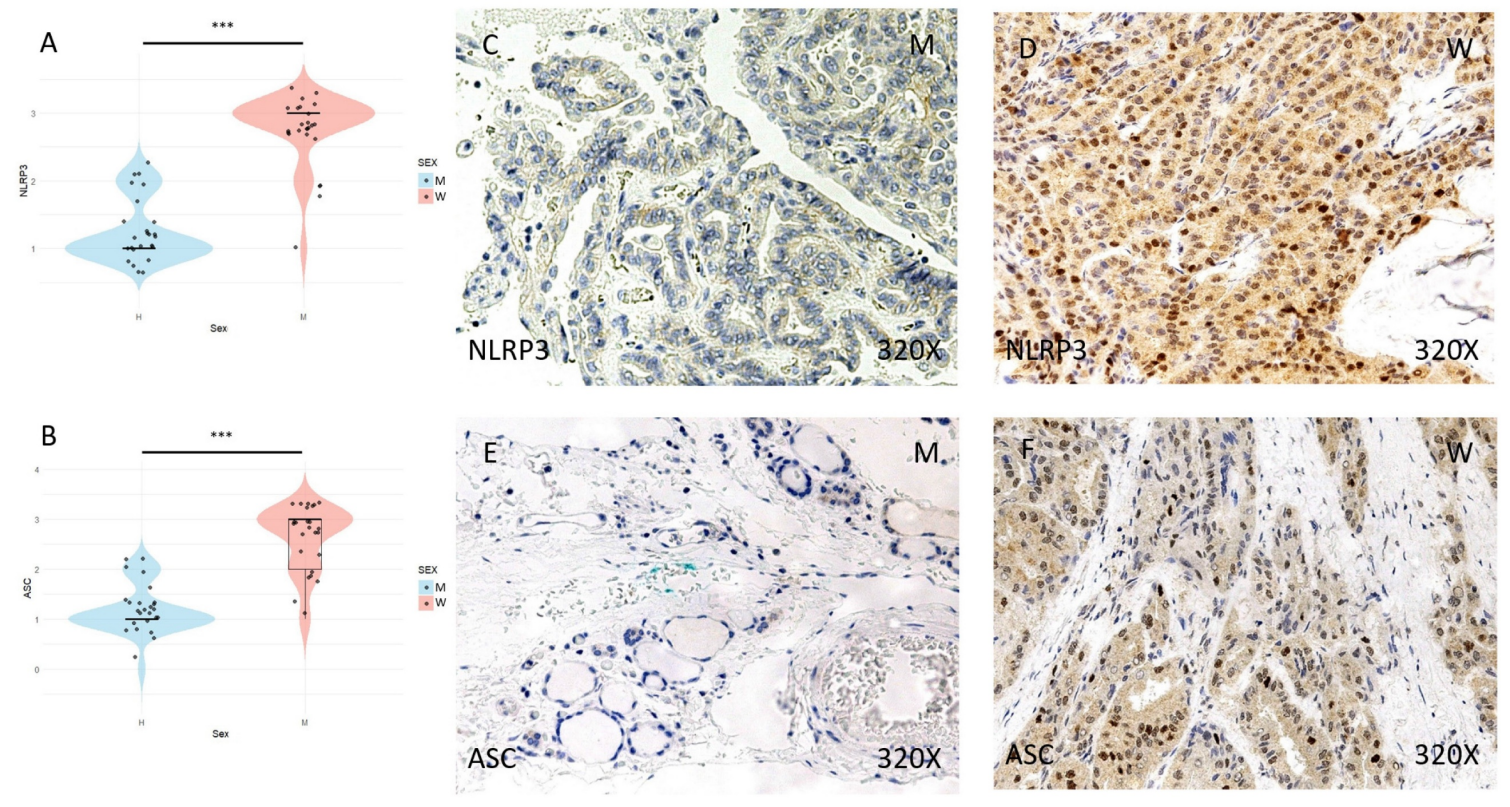
** (A)** and **(B)** Distribution of NLRP3 inflammasome marker expression (median in women: 3; median in men: 1; p-value = 1.662e-09) and ASC (median in women: 3; median in men: 1; p-value = 2.778e-08) in papillary thyroid carcinoma samples by sex, represented by a violin plot. **(C-F)** Images of immunohistochemical expression of NLRP3 and ASC, showing increased protein expression in women. 320x magnification. Significance levels: p<0.05 (*), p<0.01 (**), p<0.001(***).

**Figure 5:**
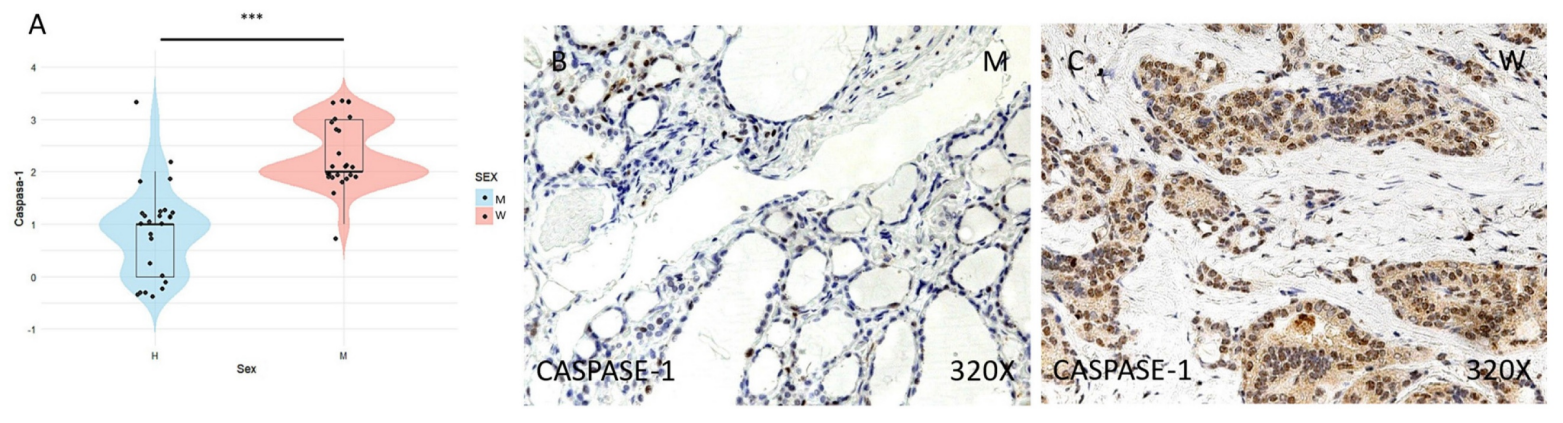
** (A)** Distribution of CASPASE-1 inflammasome marker expression (median in women: 2; median in men: 1; p value=1.518e-07) in papillary thyroid carcinoma samples by sex, represented by a violin plot. **(B-C)** Images of CASPASE-1 expression at the immunohistochemical level, showing increased protein expression in women. 320x magnification. Significance levels: p<0.05 (*), p<0.01 (**), p<0.001(***).

**Figure 6:**
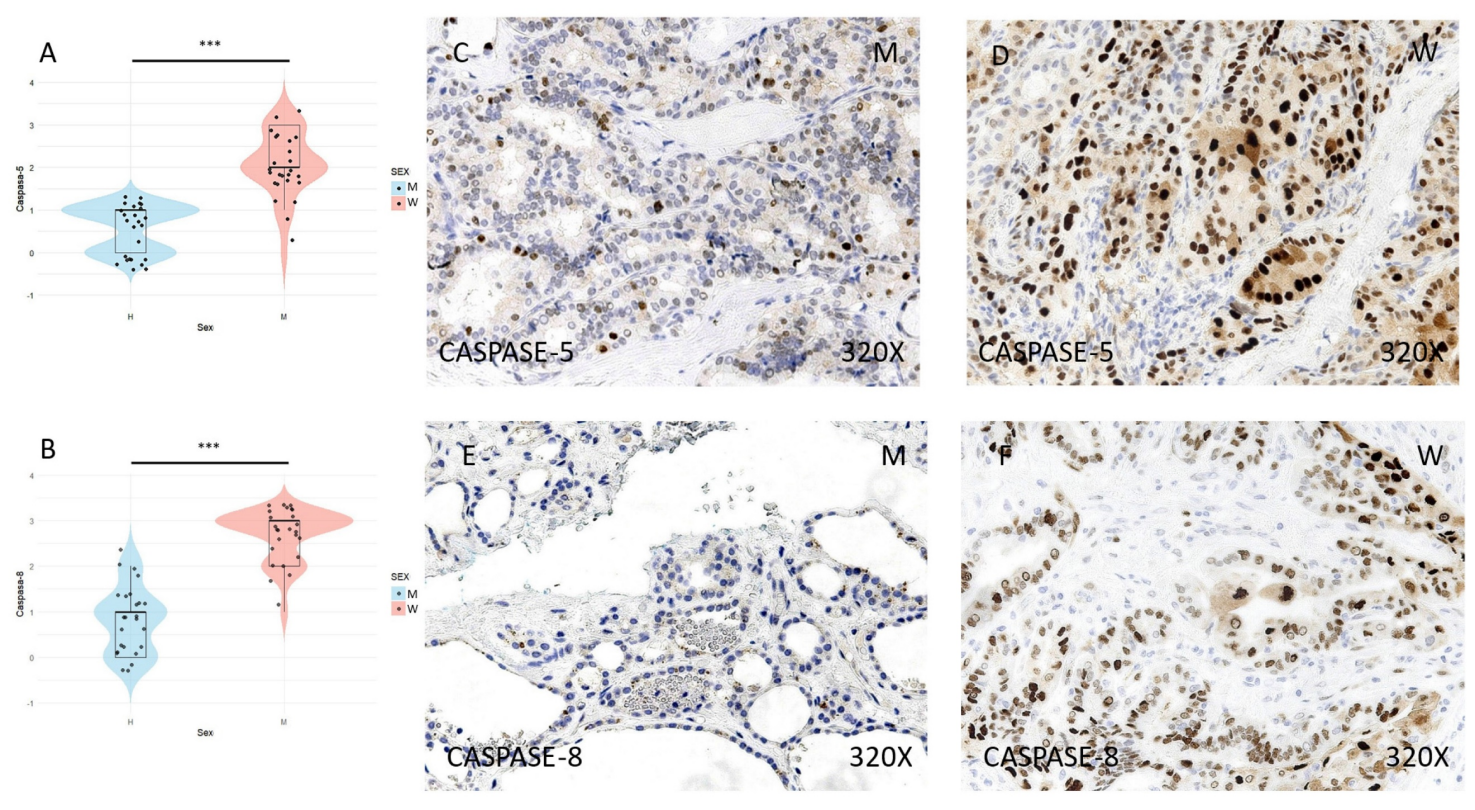
**(A)** and **(B)** Distribution of inflammasome marker expression (median in women: 2 and median in men: 1; p-value: 4.855e-08) and CASPASE-8 (median in women: 3; median in men: 1; p-value=4.656e-09) in papillary thyroid carcinoma samples according to sex, represented by a violin plot. **(C-F)** Images of *i*mmunohistochemical expression of CASPASE-5 and CASPASE-8, showing increased protein expression in women. 320x magnification. Significance levels: p<0.05 (*), p<0.01 (**), p<0.001(***).

**Figure 7:**
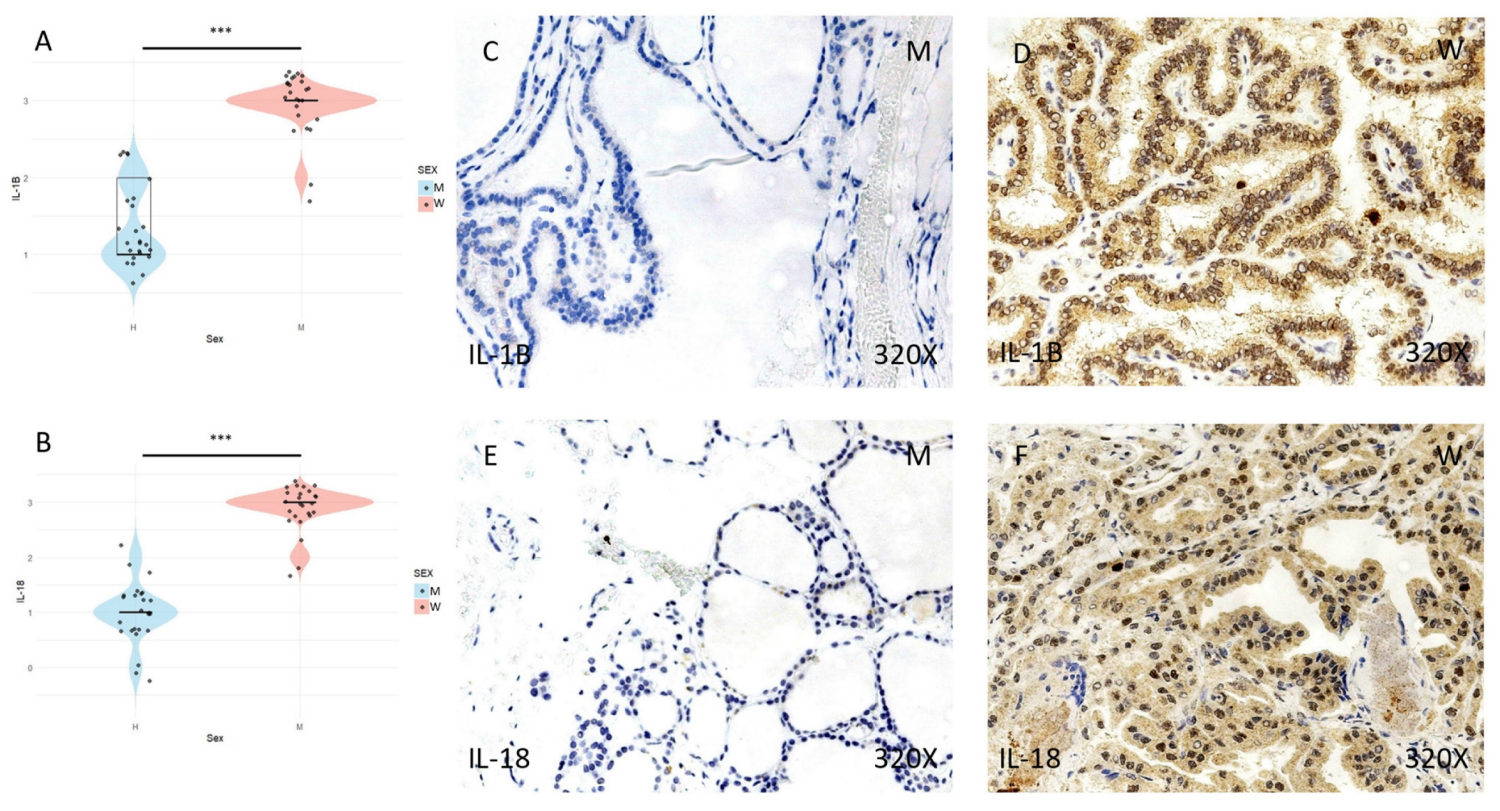
**(A)** and **(B)** Distribution of the expression of IL-1β inflammasome markers (median in women: 3; median in men: 1; p value=1.813e-10) and IL-18 (median in women: 3 and median in men: 1; p value=1.195e-10) in papillary thyroid carcinoma samples according to sex, represented by a violin plot. **(C-F)** Images of immunohistochemical expression of IL-1β and IL-18, showing increased protein expression in women. 320x magnification. Significance levels (*) = p<0.05, (**) = p<0.01, (***) = p<0.001.

Finally, the expression of the inflammasome markers NLRP3, ASC, CASPASE-1, CASPASE-5, CASPASE-8, IL-1β, and IL-18 was studied using immunohistochemical techniques, showing an increase in the tumor tissue of women compared to that of men (**Figures 4 to 7** (**C-F**)). After quantification, this difference was significant in all cases, namely NLRP3 (median in women: 3; median in men: 1; p-value=1.662e-09), ASC (median in women: 3; median in men: 1; p-value=2.778e-08), caspase-1 (median in women: 2; median in men: 1; p value=1.518e-07), caspase-5 (median in women: 2 and median in men: 1; p value: 4.855e-08), caspase-8 (median in women: 3; median in men: 1; p value=4.656e-09), IL-1β (median in women: 3, median in men: 1; p value=1.813e-10), IL-18 (median in women: 3 and median in men: 1; p value=1.195e-10) (**Figures 4 to 7 (A-B)**).

### 4.4 The study of correlations between markers of proliferation, aging, ferroptosis, and inflammasome expression reveals differential patterns depending on sex

**Figure 8:**
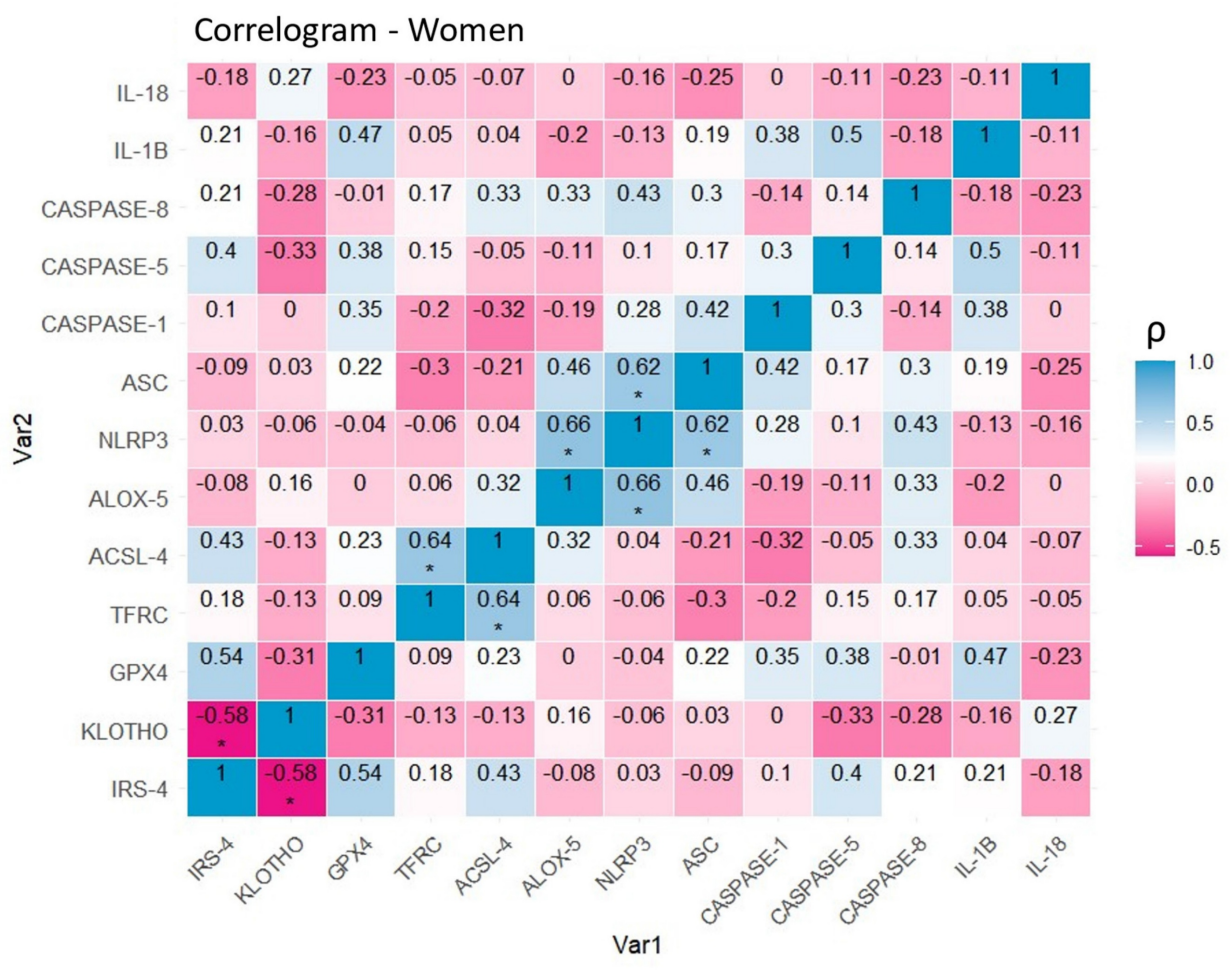
Correlation of variables in the group of women with papillary thyroid carcinoma. Correlation matrix and heat map of the molecular markers included in the study. Note: The graph shows Spearman's coefficients with their corresponding degrees of significance. The diagonal represents the perfect correlation (1), that of each protein with itself. Warm colors represent negative correlations (-0.58,0) or inversely proportional in this specific case, while cool colors represent positive correlations (0,0.66) or directly proportional in this specific case. Significance levels: p<0.05 (*), p<0.01 (**), p<0.001(***).

After stratification by sex, correlation matrices were developed to clarify molecular expression relationships between pairs of markers. In the group of female patients with papillary thyroid carcinoma (**Figure 8**), a positive correlation was observed between the ferroptosis markers TFRC and ACSL-4 (0.64, p<0.05 (*)), as well as between the inflammasome-related proteins NLRP3 and ASC (0.62, p<0.05 (*)). Representatives of both processes also show evidence of interrelation, as evidenced by the positive correlation between NLRP3 and ALOX-5 (0.66, p<0.05 (*)). Finally, a negative linear relationship between IRS-4 and KLOTHO (- 0.58, p<0.05 (*)) was also established.

**Figure 9:**
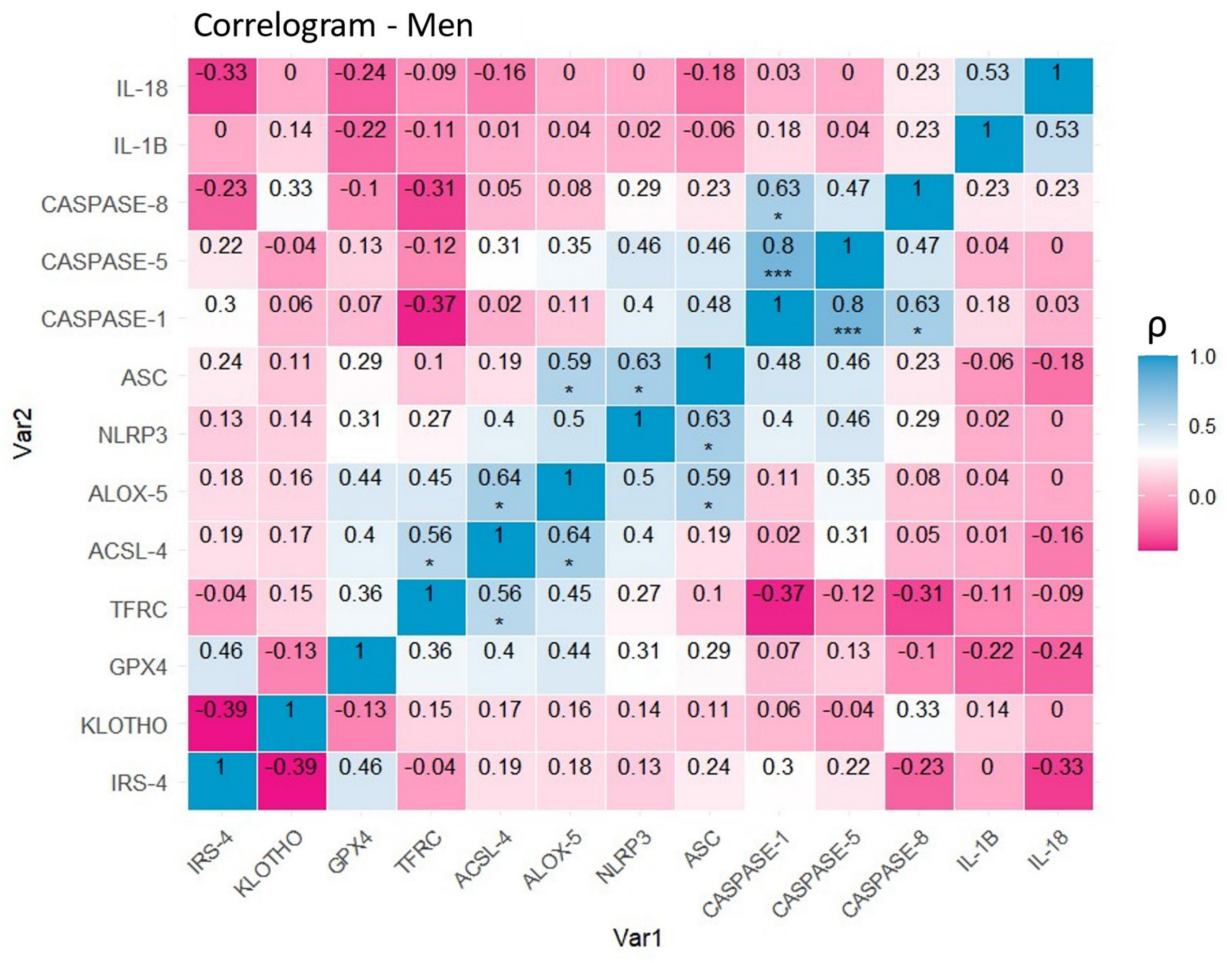
Correlation of variables in the group of men with papillary thyroid carcinoma. Correlation matrix and heat map of the molecular markers included in the study. Note: The graph shows Spearman's coefficients with their corresponding degrees of significance. The diagonal represents the perfect correlation (1), that of each protein with itself. Warm colors represent negative correlations (-0.39,0) or inversely proportional in this specific case, while cool colors represent positive correlations (0,0.8) or directly proportional in this specific case. Significance levels: p<0.05 (*), p<0.01 (**), p<0.001(***).

Regarding the correlograms of male patients (Figure 9), positive correlations were observed between CASPASE-1 and CASPASE-5 (0.8, p<0. 001 (***)), CASPASE-1 and CASPASE-8 (0.63, p<0.05 (*)), as well as between the NLRP3 and ASC inflammasome-related proteins (0.63, p<0.05 (*)). Similarly, there is evidence of a positive correlation between the pairs of ferroptotic markers TFRC and ACSL-4 (0.56, p<0.05 (*)), and ALOX-5 and ACSL-4 (0.64, p<0.05 (*)). Once again, the results illustrate a significant correlation between the ferroptotic protein ALOX-5 and inflammasome proteins, in this case, ASC (0.59, p<0.05 (*)).

## 5. Discussion

In this study, we observed differential expression between men and women with TPC of multiple ferroptosis markers (GPX4, ALOX-5, ACSL4, and TFRC), the NLRP3 inflammasome (NLRP3, ASC, caspase 1, caspase 5, caspase 8, IL-1β, and IL-18) and molecules linked to cell proliferation (IRS-4) and with antitumor functions (KLOTHO). More specifically, women show an increase in markers of ferroptosis, NLRP3 inflammasome, and IRS-4, while men show higher levels of KLOTHO. These results will be analyzed in the discussion, structured around these four axes, with the aim of delving deeper into the molecular mechanisms underlying these sex differences and evaluating their possible impact on carcinogenesis, prognosis, and therapeutic strategies for TPC.

### 5.1. Ferroptosis

The term ferroptosis was proposed in 2012 by Dixon *et al*. and refers to a type of iron-dependent regulated cell death characterized by persistent lipid peroxidation leading to the accumulation of lipid-derived reactive oxygen species (ROS), resulting in lethal membrane damage and cell death due to altered ion fluxes, water influx, and biophysical effects 36. Ferroptosis is intrinsically linked to proliferation, progression, metastasis, and therapeutic response in various types of cancer, playing a dual role, as it can both promote and inhibit these processes 37. The TPC has demonstrated the relevance of ferroptosis as a mechanism involved in carcinogenesis, as well as its potential translational value in the development of diagnostic, prognostic, and therapeutic strategies 38-40.

In this study, we observed for the first time that women with TPC show a significant increase in the ferroptosis markers analyzed (GPX4, ALOX5, ACSL4, and TFRC) compared to men. GPX4 is a key negative regulator of ferroptosis, transforming harmful lipid peroxides into harmless lipid alcohols, thereby limiting the process of lipid peroxidation 17,41-43. Previous studies have shown that GPX4 is highly overexpressed in thyroid cancer. More specifically, this component is significantly associated with advanced clinical stages (T3-T4 and pathological stages III-IV) and is listed as an independent risk factor for overall patient survival 14,44,45. For this reason, pharmacological modulation of GPX4 has been proposed as a promising therapeutic target in thyroid cancer and other types of tumors 14,45. *In vitro* experiments have shown that GPX4 silencing not only suppresses cell proliferation but also induces both apoptosis and ferroptosis in TPC cell lines 46. The possible mechanisms that could explain the differences in GPX4 expression in the TPC between men and women have not yet been explored. However, previous studies have shown that estrogen can regulate GPX4 expression in different cell types, such as osteoblasts 18. Future studies should focus on analyzing the underlying causes and consequences of GPX4 alteration in men and women with TPC.

ALOX5 or arachidonate 5-lipoxygenase is an enzyme belonging to the lipoxygenase family. It is responsible for the oxidation of polyunsaturated fatty acids (PUFAs), especially arachidonic acid, thereby influencing the immune response and lipid peroxidation 36,47. Furthermore, ALOX5 uses iron as a catalytic cofactor, and its enzymatic activity is enhanced by an increase in the availability of this element 48, thus establishing itself as a key marker of ferroptosis. ALOX5 has been found to be involved in several types of tumors, including bladder cancer 49, pancreatic cancer^50^ or lung cancer 51.

ALOX5 is overexpressed in TPC, correlating with an invasive phenotype 52. Its involvement in the induction of matrix metalloproteinase-9 (MMP-9) suggests a key role in extracellular matrix remodeling, which has prompted its study as a diagnostic and prognostic biomarker in thyroid cancer 53-55. Although intersex differences in the levels of this enzyme still need to be explored in greater depth, Mirra *et al*. suggested that women have a higher abundance of two polymorphisms (rs2029253 and rs2115819) that alter the transcriptional activity of ALOX-5 56. In this regard, it would be interesting for future studies to explore this polymorphism and other possible explanations for the variations found in our study.

ACSL4 or acyl Co-A synthase 4 is a key enzyme in fatty acid metabolism, increasing the incorporation of PUFAs into phospholipids, making them more susceptible to oxidation and therefore to the process of ferroptosis 57,58. ACSL4 exhibits specific patterns that position it as a promising biomarker in thyroid oncology 59,60. Pan-cancer analysis studies have revealed a notable overexpression of ACSL4 in thyroid cancer cell lines, according to data from the Cancer Cell Line Encyclopedia 61. However, its potential as a biomarker in this type of tumor has not yet been fully objectified. Similarly, specific analyses of gender differences in expression in thyroid cancer show limitations in the current literature, although studies in other types of cancer have established that ACSL4 expression is inversely associated with hormone receptors such as estrogen and androgen receptors, suggesting a potential hormonal regulation mechanism that could influence the differences observed between men and women in the incidence and aggressiveness of thyroid cancer 62.

The transferrin receptor (TFRC) is a membrane protein found on the cell surface that binds to iron-loaded transferrin, facilitating the uptake of this element into the cell. Thus, overexpression of TFRC promotes intracellular iron accumulation, triggering the process of ferroptosis 63. TFRC is significantly overexpressed in thyroid carcinomas compared to benign thyroid tissue, with significantly higher protein levels in primary papillary carcinoma, metastatic carcinoma, and anaplastic carcinoma, thus acting as an important diagnostic biomarker 64. Similarly, overexpression of TFRC has been linked as a biomarker of poorer prognosis in TPC 38,39. As for gender differences, although specific studies on the thyroid are scarce, pan-cancer analyses indicate that TFRC expression may correlate with sex in various tumors; for example, in low-grade gliomas, higher expression was observed in women (p=0.043) 65. In addition, transcriptomic studies from The Cancer Genome Atlas (TCGA) show that TFRC tends to be higher in women with thyroid cancer 66, suggesting a possible hormonal regulatory effect on its transcription and a role in the disparity in incidence and aggressiveness between genders. However, targeted research is needed to conclusively confirm intersexual differences in TFRC expression in thyroid cancer and its clinical relevance.

In conclusion, our findings reinforce the central role of ferroptosis in the pathophysiology of TPC, highlighting a differential expression by sex in the main markers studied (GPX4, ACSL4, ALOX5, and TFRC), with significant overexpression in women. These results suggest the possible involvement of hormonal factors in the regulation of these genes and open new lines of research to better understand the molecular basis of gender differences in this type of cancer. Furthermore, the identification of these markers as potential therapeutic targets offers a promising field for the development of personalized strategies in the treatment of TPC.

### 5.2. NLRP3 inflammasome

The NLRP3 inflammasome multiprotein complex is part of the innate immune system and consists of a protein that acts as a sensor for danger or infection signals (NLRP3), an adapter (ASC, also known as PYCARD), and an effector protein (caspase-1) 67. NLRP3 has three domains: a pyrin domain (PYD) that initiates assembly by binding to ASC, a NACHT domain that is activated using ATP and enables oligomerization, and an LRR domain that negatively regulates the process 68. When NLRP3 is activated by canonical pathways, it binds to ASC through PYD-PYD interactions, which in turn recruits pro-caspase-1 through the CARD domains they share, allowing caspase-1 to be activated. The activation of caspase-1 leads to the cleavage of pro-IL-1β, pro-IL-18, and gasdermin D (GSDMD), whose N-terminal fragment forms transmembrane pores that allow the release of IL-1β and IL-18 in their active form 68. One of the consequences of NLRP3 inflammasome activation is pyroptosis, a type of highly inflammatory regulated cell death; although its activation is also implicated in other types of cell death such as apoptosis, necroptosis, PANoptosis, and ferroptosis itself 69. In addition, recent studies have demonstrated non-canonical pathways of NLRP3 complex activation involving caspases 5 and 8 21,70.

The activation of the NLRP3 inflammasome occurs in two phases: first, a priming phase in which stimuli such as TLRs, TNF, or IL-1β induce the production of NLRP3 and pro-IL-1β through the NF-κB factor; and second, an activation phase, where NLRP3 detects intracellular signals of damage or infection (DAMP) 71. This process is crucial for promoting acute inflammatory responses necessary to fight infections or repair injuries; however, its dysregulation over time can trigger chronic inflammatory processes and pathologies 72. Inflammation is one of the hallmarks of cancer recognized by Hannahan and Weinberg 73. Scientific literature highlights the relevance of different types of inflammasomes in the carcinogenesis process in different types of tumors 74. As for the NLRP3 inflammasome, it has been determined that it plays a dual role and can act both to facilitate tumor progression and to suppress it, depending on the context 67. To date, evidence of the role of the NLRP3 inflammasome in TPC is limited. Studies have shown how NLRP3 and other inflammasomes are involved in the pathogenesis of autoimmune thyroiditis 75 standing out as a potential therapeutic target in this context 76

For the first time, our results suggest a possible role for the NLRP3 inflammasome in the carcinogenesis of TPC, and that there may also be a differential role depending on sex. In general terms, sex hormones are considered to modulate immune system activity differentially: estrogens tend to enhance the immune response, while progesterone and androgens, such as testosterone, act predominantly as immunosuppressants 77. Several studies suggest that female sex hormones, especially estrogen, enhance the activation of the NLRP3 inflammasome in hormone-sensitive tumors such as papillary thyroid carcinoma. Estradiol binds to the estrogen receptor β (ERβ) present in malignant thyroid cells, which increases the transcription of NLRP3 and its components (ASC and caspase-1), promoting the maturation of IL-1β and IL-18 and generating a proinflammatory microenvironment that promotes tumor proliferation 23,78. In contrast, testosterone exerts a direct inhibitory effect on NLRP3, reducing the production of mitochondrial reactive oxygen species (mROS) necessary for inflammasome assembly and attenuating the inflammatory response 79,80. This hormonal duality explains the gender bias observed in the overactivation of the NLRP3 inflammasome in women with PC, where estrogen dominance reinforces the inflammatory signal and contributes to tumor progression.

This could explain the differences observed in this study in the expression of molecules involved in the activation of the NLRP3 inflammasome complex in men and women, although further research is needed to investigate the possible underlying mechanisms responsible for these differences.

### 5.3. KLOTHO

KLOTHO is a protein with key functions in cell protection and metabolic regulation. It acts as an antioxidant, inhibits apoptosis and fibrosis, and promotes angiogenesis and vascular health. In addition, it regulates various metabolic pathways involved in energy balance, as well as glucose and lipid homeostasis, contributing to cell protection and healthy aging 24.

KLOTHO is considered an important tumor suppressor in various solid and hematological cancers, interfering with key cell signaling pathways. For example, KLOTHO is responsible for inhibiting different intracellular pathways related to cell proliferation and growth, such as PI3K/AKT, MAPK/ERK, WNT/β-catenin, and TGFβ, while also activating regulated cell death pathways 81,82. In most cancers, KLOTHO is underexpressed or silenced, and its dysregulation serves as a highly relevant prognostic biomarker 26,27,83-85.

In TPC, KLOTHO shows significantly reduced expression when compared to benign thyroid tissue. Immunohistochemical analysis shows that benign tumors (nodular hyperplasia and follicular adenoma) maintain positive immunoreactivity in the follicular epithelium, while in differentiated cancers (follicular and papillary) expression is decreased, thus suggesting KLOTHO as a critical mechanism involved in thyroid tissue carcinogenesis 86. In TPC cell lines, KLOTHO acts as a tumor suppressor by inhibiting the Wnt/β-catenin pathway and cyclin D1, reducing proliferation and increasing apoptosis in follicular thyroid cancer cell lines, while its silencing promotes cell growth 28,87. In addition, KLOTHO regulates the expression of stanniocalcin-1 (STC1), a tumor marker, where high levels of KLOTHO are inversely associated with low levels of STC1, and treatment with recombinant human STC1 attenuates KLOTHO-induced cell growth inhibition 87. Its value as a prognostic biomarker is established by the inverse correlation between low KLOTHO expression and more advanced stages of differentiated thyroid carcinoma 88.

Our study supports the role of KLOTHO in the carcinogenesis process of TPC, suggesting a differential role of this protein between men and women. This is supported by studies that have found higher expression of KLOTHO in male mice than in female mice 29. On the other hand, research on serum KLOTHO and thyroid hormones shows significant differences according to gender. In men, the association between KLOTHO and free T3 is weaker than in women, but the correlations with total T3, total T4, and their ratios are significant only in men 89. This suggests that hormonal regulation by estrogen may be stronger than the influence of KLOTHO in women, while men may be more susceptible to the effects of KLOTHO on thyroid function. These differences may help explain the variations in the incidence and aggressiveness of thyroid cancer between genders, although further studies are needed to confirm these specific differences in KLOTHO expression.

### 5.4. IRS-4

Insulin receptor substrate 4 (IRS-4) is part of a family of cytoplasmic adapter proteins called IRS that transmit signals from transmembrane receptors such as the insulin receptor (IR) and the insulin-like growth factor 1 receptor (IGF1R) to intracellular signaling pathways [90,91]Although it shares some structural domains with other members of this family, such as the PH domain (pleckstrin homology) and the PTB domain (phosphotyrosine binding domain), IRS-4 has unique characteristics that allow it to interact with different kinases and intracellular adapter proteins 92.

Recently, the role of IRS-4 in cancer has been investigated, identifying its involvement in the activation of two key pathways for tumor proliferation: the PI3K/AKT pathway and the MAPK (Ras/Raf/MEK/ERK) pathway. The PI3K/AKT pathway is involved in processes such as cell proliferation, growth, cytoskeletal remodeling, autophagy, and apoptosis 93, while the MAPK pathway regulates proliferation, cell differentiation, apoptosis, and stress response 94.

IRS-4 can activate the PI3K/AKT pathway in several ways, including conventional mechanisms such as the interaction of IRS-4 with kinases such as FER and BRK and adaptors such as CRKL (proteins that are frequently overexpressed in various types of tumors such as ovarian and breast cancer) 95-97. IRS-4 can also induce constitutive activation of AKT in the absence of stimuli 98 or it can interact with the BMPRII receptor, which also triggers the activation of AKT 99. Furthermore, through the formation of complexes with adapter proteins, IRS-4 facilitates the sequential activation of Raf, MEK, and ERK kinases, which are essential for cell survival and tumor progression 94. It has also been shown that IRS-4 can bind directly to C-Raf, reinforcing its role in activating this pathway 100.

These various pathways converge in the sustained activation of pro-cancerous cellular mechanisms. Studies demonstrating the aberrant activation of IRS-4 in multiple tumor types, as well as its contribution to tumor development and progression, position IRS-4 as a key molecule in cancer biology 30. However, the role of IRS-4 in TPC has not yet been studied. The expression of IRS-4 in the thyroid is of great physiological and clinical relevance. Mutations that cause loss of function in IRS4 lead to X-linked congenital central hypothyroidism in males, characterized by insufficient TSH secretion and low levels of free T4, without structural abnormalities in the hypothalamic-pituitary-thyroid axis 101. However, in mouse models with Irs4 deletions, no alterations in TSH, TRH, or thyroxine levels are observed, suggesting that other IRS proteins may compensate for its function 102. In addition, genetic association studies have identified variants in the IRS4 locus that are associated with an increased risk of thyroid disorders and hypothyroidism in humans 103. Since IRS4 is an X-linked gene, its mutations and regulation may be influenced by sex, suggesting a differential role in thyroid function between men and women 101 Together, these data highlight the importance of investigating the role of IRS-4 in TPC, considering possible sex differences that could impact diagnosis and treatment.

### 5.4. Correlation differences between sexes

Analysis of correlations between markers based on sex reveals distinctive molecular patterns in papillary thyroid carcinoma. In female patients, positive associations between ferroptosis markers (TFRC and ACSL4) and inflammasome components (NLRP3 and ASC) stand out, as well as cross-correlations between both processes, such as NLRP3 and ALOX5, suggesting a possible functional synergy between inflammation and ferroptotic cell death. In addition, the negative correlation between IRS-4 and KLOTHO reinforces the hypothesis of an inverse relationship between proliferation and tumor suppression modulated by sex. In males, although significant correlations are also evident between inflammasome proteins (CASPASE-1, -5, -8) and between ferroptosis markers (TFRC, ACSL4, ALOX5), the associations appear to be more localized within each functional pathway. However, the correlation between ALOX5 and ASC suggests that there may also be a relevant interaction between inflammation and ferroptosis in men. These findings support the existence of sex-differentiated molecular networks, which could have important implications for understanding tumor biology and developing personalized therapeutic strategies.

### 5.5. Limitations

Despite the results obtained in this study, it is important to note a number of limitations that may have influenced the findings. One of the main limitations is the absence of thyroid samples from patients without PTC, i.e., the lack of a healthy control group. Furthermore, the technique used to measure the expression levels of the selected markers was exclusively immunohistochemistry, which, although useful for detecting differences in the localization and relative levels of proteins, does not allow for precise quantification or analysis of the underlying molecular mechanisms that explain the differences detected. Complementary techniques such as real-time PCR or Western blot would have allowed for a more quantitative and functional validation of the results. In addition, the study focused solely on the histological type of the tumor, selecting only PTC samples, without taking into account the tumor stage or the treatment received by the patients, factors that could have influenced the expression of the markers analyzed. Similarly, although the sample size allowed significant differences to be detected, it may not be sufficiently representative of the entire TPC population, thus limiting the generalizability of the results. It is also important to consider the biological heterogeneity of tumor tissue and the fact that this is a cross-sectional analysis based on a single sample collection time point, which prevents the establishment of temporal or causal relationships. Finally, the exclusive use of immunohistochemistry may introduce some variability in the interpretation, due to the subjectivity in the assignment of scores and the dependence on the quality of the antibodies used.

## 6. Conclusions

This study investigated the differential expression by sex of different biomarkers linked to certain hallmarks of cancer. More specifically, immunohistochemistry has shown that samples from women with TPC present increased expression of ferroptosis markers (GPX4, ALOX5, ACSL4, and TFRC), NLRP3 inflammasome markers (NLRP3, ASC, caspase-1, caspase-5, caspase-8, IL-1β, and IL-18) and cell proliferation (IRS-4) markers, compared to samples from men, in which increased expression of the tumor suppression marker (KLOTHO) was observed. These findings confirm our hypothesis that clinical-biological variability between sexes is associated with differences in changes in the expression of molecular mediators of ferroptosis, inflammation, proliferation, and tumor suppression. The activation of the different pathways could reflect an adaptive cellular response modulated by the action of the specific hormonal environment of each sex. However, further research is needed, with a larger sample size and a mechanistic approach, to explore in depth the specific mechanisms of these molecular pathways and their interaction with hormonal factors, as well as to develop effective and specific therapeutic interventions.

## Figures and Tables

**Table 1:** Primary and secondary antibodies and their dilutions

Antigen	Dilution	Supplier	Protocol specifications
IRS-4	1:500	Thermo Fisher Scientific- PA5-117329	Pre-incubation with TRIS-EDTA buffer pH=9 and incubation with 0.1% TTX (Trotón x100 in TBS) for 5 min
KLOTHO	1:100	Abcam (ab181373)	-
GPX4	1:100	Abcam (ab125066)	10 mM sodium citrate, pH=6 before incubation with blocking solution
TFRC	1:500	Abcam (ab18550)	EDTA pH=9 before incubation with blocking solution
ACSL-4	1:100	Abcam (ab155282)	100% Triton at 0.1% in PBS, 10 min, before incubation with blocking solution
ALOX-5	1:250	Abcam (ab169755)	100% Triton at 0.1% in PBS, 10 min, before incubation with blocking solution
NLRP3	1:500	Abcam (ab263899)	Sodium citrate 10 mM pH=6 before incubation with blocking solution
ASC	1:250	Abcam (ab283684)	100% Triton at 0.1% in PBS, 10 min, before incubation with blocking solution
CASPASE-1	1:500	Abcam (ab62698)	EDTA pH=9 before incubation with blocking solution
CASPASE-5	1:100	Abcam (ab40887)	10 mM sodium citrate, pH=6 before incubation with blocking solution
CASPASE-8	1:250	Abcam (ab25901)	100% Triton at 0.1% in PBS, 10 min, before incubation with blocking solution
IL-1β	1:50	Abcam (ab283818)	-
IL-18	1:50	Abcam (ab243091)	-
IgG (Rabbit)	1:300	RG-96; Sigma-Aldrich, St. Louis, MI, EE. UU.	-
IgG (Goat)	1:100	GT-34/B3148; Sigma-Aldrich	-
IgG (Mouse)	1:300	F2012/045K6072; Sigma-Aldrich	-
